# Randomized phase III trial of APF530 versus palonosetron in the prevention of chemotherapy-induced nausea and vomiting in a subset of patients with breast cancer receiving moderately or highly emetogenic chemotherapy

**DOI:** 10.1186/s12885-016-2186-4

**Published:** 2016-02-26

**Authors:** Ralph Boccia, Erin O’Boyle, William Cooper

**Affiliations:** Center for Cancer and Blood Disorders, 6410 Rockledge Drive #660, Bethesda, MD 20819 USA; FibroGen, Inc, 409 Illinois Street, San Francisco, CA 94158 USA; TFS, Inc, 212 Carnegie Center, Suite 208, Princeton, NJ 08540 USA

**Keywords:** Breast cancer, Antiemetics, Granisetron, Sustained-release preparations, Subcutaneous injections

## Abstract

**Background:**

APF530 provides controlled, sustained-release granisetron for preventing acute (0–24 h) and delayed (24–120 h) chemotherapy-induced nausea and vomiting (CINV). In a phase III trial, APF530 was noninferior to palonosetron in preventing acute CINV following single-dose moderately (MEC) or highly emetogenic chemotherapy (HEC) and delayed CINV in MEC (MEC and HEC defined by Hesketh criteria). This exploratory subanalysis was conducted in the breast cancer subpopulation.

**Methods:**

Patients were randomized to subcutaneous APF530 250 or 500 mg (granisetron 5 or 10 mg) or intravenous palonosetron 0.25 mg during cycle 1. Palonosetron patients were randomized to APF530 for cycles 2 to 4. The primary efficacy end point was complete response (CR, no emesis or rescue medication) in cycle 1.

**Results:**

Among breast cancer patients (*n* = 423 MEC, *n* = 185 HEC), > 70 % received anthracycline-containing regimens in each emetogenicity subgroup. There were no significant between-group differences in CRs in cycle 1 for acute (APF530 250 mg: MEC 71 %, HEC 77 %; 500 mg: MEC 73 %, HEC 73 %; palonosetron: MEC 68 %, HEC 66 %) and delayed (APF530 250 mg: MEC 46 %, HEC 58 %; 500 mg: MEC 48 %, HEC 63 %; palonosetron: MEC 52 %, HEC 52 %) CINV. There were no significant differences in within-cycle CRs between APF530 doses for acute and delayed CINV in MEC or HEC in cycles 2 to 4; CRs trended higher in later cycles, with no notable differences in adverse events between breast cancer and overall populations.

**Conclusions:**

APF530 effectively prevented acute and delayed CINV over 4 chemotherapy cycles in breast cancer patients receiving MEC or HEC.

**Trial registration:**

Clinicaltrials.gov identifier: NCT00343460 (June 22, 2006).

## Background

Chemotherapy-induced nausea and vomiting (CINV) is common in patients receiving chemotherapy and, if untreated, can lead to numerous adverse consequences, including metabolic imbalances, anorexia, dehydration, and poor compliance with therapy [1, 2]. Furthermore, once CINV is experienced, anticipatory nausea and vomiting may ensue during later cycles of chemotherapy [3]. Uncontrolled CINV can also have economic effects, including an increase in unplanned office visits, hospitalizations, or hydration therapy [4].

Chemotherapeutic agents were first classified by Hesketh according to their emetogenic potential in the absence of antiemetic prophylaxis, with the risk of CINV being 31–90 % in patients receiving moderately emetogenic chemotherapy (MEC) and > 90 % in patients receiving highly emetogenic chemotherapy (HEC) [5, 6]. Several antiemetic guidelines are available for the prevention of CINV in both the acute phase (occurring within 24 h after chemotherapy) and the delayed phase (occurring 24–120 h after chemotherapy). Current antiemetic guidelines are similar with respect to the prevention of CINV after HEC, recommending a combination of a 5-hydroxytryptamine type 3 (5-HT_3_) receptor antagonist, dexamethasone, and a neurokinin 1 (NK-1) antagonist. For the prevention of CINV after MEC, guidelines differ somewhat depending on the chemotherapy regimen, but generally a 5-HT_3_ antagonist plus dexamethasone (plus an NK-1 antagonist for some patients) is recommended [7–9]. However, despite the availability of multiple antiemetic agents and treatment guidelines, there is still an unmet need for adequate prevention of delayed CINV in patients receiving MEC and HEC.

The effects of breast cancer and its treatment on quality of life in patients with breast cancer are well documented, and several quality of life instruments include measures of nausea and/or vomiting [10–12]. Most patients with breast cancer who are treated with chemotherapy receive, at minimum, a regimen classified as MEC, so many are at risk for experiencing CINV unless they receive adequate preventive therapies [13–15]. Combination chemotherapy consisting of an anthracycline (doxorubicin or epirubicin) and cyclophosphamide (AC) is commonly used for the treatment of breast cancer, with or without other agents. This combination was historically classified as MEC, but AC-based regimens were recently reclassified as highly emetogenic in the American Society of Clinical Oncology (ASCO) antiemetic guidelines [8].

Granisetron is a first-generation 5-HT_3_ receptor antagonist commonly used to treat CINV, but its short half-life (8 h) makes it unsuitable for the effective prevention of delayed CINV [16]. APF530 is a novel formulation of 2 % granisetron and a bioerodible tri(ethylene glycol) poly (orthoester) (TEG-POE) polymer that is designed to provide slow, controlled hydrolysis, resulting in slow and sustained release of granisetron for the prevention of both acute and delayed CINV associated with MEC and HEC [17]. In a previous trial, patients receiving MEC or HEC also received subcutaneously (SC) an abdominal injection of APF530 250, 500, or 750 mg (5, 10, or 15 mg granisetron, respectively). The half-life (t_1/2_) of granisetron administered in this formulation was ~24–34 h, time to maximum plasma concentration (t_max_) was ~24 h, and APF530 elicited sustained therapeutic granisetron concentrations over 168 h [18]. Subsequently, a phase III noninferiority trial (Clinicaltrials.gov identifier: NCT00343460) was conducted to compare the efficacy and safety of 2 doses of APF530 (250 mg and 500 mg) with the approved dose of palonosetron (0.25 mg intravenously [IV]) for the prevention of acute and delayed CINV following single-day administration of MEC or HEC in patients with cancer. APF530 was found to be noninferior to palonosetron in the control of acute CINV in patients receiving MEC or HEC and in the prevention of delayed CINV in patients receiving MEC; however, it did not demonstrate superiority over palonosetron in delayed CINV with HEC [19]. In this post hoc analysis of the phase III trial, we review the subpopulation of patients who had breast cancer.

## Methods

Details of the study design and methodology have been presented elsewhere [19]; a brief overview is presented here.

### Study design

This prospective, multicenter, randomized, double-blind, double-dummy, parallel-group phase III trial (NCT00343460) was approved by the Institutional Review Board or Independent Ethics Committee at the following centers: Anniston Oncology, PC; Palo Verde Hematology Oncology – Glendale; Arizona Clinical Research Center, Incorporated; Arkansas Cancer Research Center at University of Arkansas for Medical Sciences; Pacific Cancer Medical Center, Incorporated; Southbay Oncology/Hematology Medical Group; Compassionate Cancer Care Medical Group Incorporated – Corona; Compassionate Cancer Care Medical Group Incorporated – Fountain Valley; Advanced Research Management Services, Incorporated; Kenmar Research Institute; Medical Oncology Care Associates – Orange; Eastern Connecticut Hematology and Oncology Associates; Providence Hospital; Pasco Pinellas Cancer Center – New Port Richey; Innovative Medical Research of South Florida, Incorporated; Columbus Clinic, PC; Clintell, Incorporated; Investigative Clinical Research, LLC; Cancer Center of Indiana; Family Medicine of Vincennes Clinical Trial Center; Medical Center Vincennes; Kentucky Cancer Clinic – Hazard; Kentuckiana Cancer Institute, PLLC; Hematology-Medical Oncology Associates at Central Maine Comprehensive Cancer Center; Mercy Medical Center; Center for Cancer and Blood Disorders at Suburban Hospital; Center for Clinical Research at Washington County Hospital; Northern Michigan Hospital; Regional Cancer Center at Singing River Hospital; Kansas City Cancer Centers – South; Star Hematology & Oncology; Veteran Affairs Medical Center – Buffalo; Falck Cancer Center at Arnot Ogden Medical Center; Hudson Valley Hematology-Oncology Associates – Poughkeepsie; Comprehensive Cancer Center at Pardee Hospital; Eastern North Carolina Medical Group, PLLC; Boice Willis Clinic, PA; McDowell Cancer Center at Akron General Medical Center; Gabrail Cancer Center – Canton Office; Gabrail Cancer Center – Dover Office; MedCentral – Mansfield Hospital; Signal Point Hematology Oncology Incorporated; Cancer Treatment Centers of America at Southwestern Regional Medical Center; Pottsville Cancer Clinic; Charleston Hematology Oncology Associates, PA; Julie and Ben Rogers Cancer Institute at Memorial Hermann Baptist Beaumont Hospital; Texas Cancer Clinic; Cancer Outreach Associates – Abingdon; Virginia Oncology Care, PC; Western Washington Oncology, Incorporated, PS at Western Washington Cancer Center; MultiCare Regional Cancer Center at Tacoma General Hospital; Mary Babb Randolph Cancer Center at West Virginia University Hospitals. The trial was conducted according to the Declaration of Helsinki. Patients were stratified according to the emetogenicity of their scheduled chemotherapy regimen (MEC or HEC).

### Patients

Eligible patients included adult (≥ 18 years old) men or women with histologically or cytologically confirmed malignancy who were scheduled to receive single-day MEC (Hesketh score 3 or 4) or HEC (Hesketh score 5) regimens according to then-applicable Hesketh emetogenicity criteria [5, 20]. Exclusion criteria included vomiting or more than mild nausea within 24 h prior to study drug administration, a heart rate–corrected (QTc) interval > 500 ms or representing a > 60 ms change from baseline, or any other cardiac abnormality predisposing to significant arrhythmia. All patients provided written informed consent.

### Treatment regimens

**Fig. 1:**
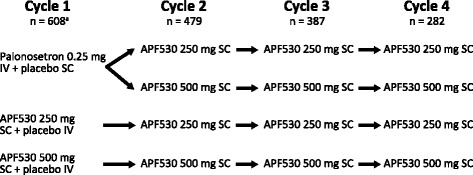
Study design. ^a^Patient numbers refer to the breast cancer modified intent-to-treat population. IV, intravenously; SC, subcutaneously

Patients were stratified according to the emetogenicity of their chemotherapy regimen (MEC or HEC) and randomized 1:1:1 to receive APF530 250 mg SC (granisetron 5 mg) plus placebo IV; APF530 500 mg SC (granisetron 10 mg) plus placebo IV; or palonosetron 0.25 mg IV plus placebo SC (Fig. 1). APF530 was administered SC in the abdomen on day 1, 30 min prior to administration of single-day MEC or HEC. On completion of cycle 1, palonosetron IV was discontinued, and patients who had received palonosetron were offered the option to remain on the study. Patients who consented were rerandomized 1:1 to receive APF530 250 mg SC or APF530 500 mg SC during cycles 2–4. Existing patients in the APF530 groups continued with the same treatment for a total of 4 chemotherapy cycles. Treatment cycles were separated by 7 to 28 days (±3 days). Protocol-specified doses of dexamethasone were administered 30–90 min prior to chemotherapy (8 mg IV for MEC, 20 mg IV for HEC). On days 2–4, oral dexamethasone 8 mg twice daily was prescribed to patients receiving HEC. Rescue medications were permitted, with the exception of granisetron, palonosetron, and aprepitant.

### Study objectives

The primary study objectives were to establish noninferiority of APF530 for the prevention of acute CINV following the delivery of MEC and HEC, compared with palonosetron 0.25 mg IV during cycle 1; noninferiority of APF530 for the prevention of delayed CINV following the delivery of MEC, compared with palonosetron 0.25 mg IV during cycle 1; and superiority of APF530 for the prevention of delayed CINV following the delivery of HEC, compared with palonosetron 0.25 mg IV during cycle 1. The primary efficacy end point was the percentage of patients achieving complete response (CR; defined as no emetic episodes and no use of rescue medications) during the acute and delayed phases of CINV in cycle 1. Secondary objectives (not reported) included evaluation of CR rates during the overall phase (0–120 h) during cycle 1 and evaluation of the rates of CR, complete control (CC; CR with no more than mild nausea), and total response (TR; CR with no nausea) across each cycle.

Adverse events (AEs; based on standard toxicity criteria) and serious AEs were assessed during each treatment cycle; assessment included type, duration, severity (mild, moderate, severe), and relationship to study drug.

### Statistical analysis

Efficacy analyses were performed separately for the MEC and HEC strata within the breast cancer subpopulation of the modified intent-to-treat (mITT) group, comprising all randomized patients who received study drug and had postbaseline efficacy data. The safety population comprised all patients who were randomized and received study drug.

Treatment group CR rates were compared using Fisher’s exact test within cycle for the breast cancer subpopulation. Quantitative variables were summarized by sample size, mean, median, standard deviation, minimum, and maximum. Qualitative variables were summarized by number and percentage of patients. Statistical significance was reached if the 2-sided *P* value was < 0.05. Comparisons between the breast cancer population and overall population were exploratory in nature and not conducted to evaluate inferiority.

## Results

### Patient characteristics

**Fig. 2:**
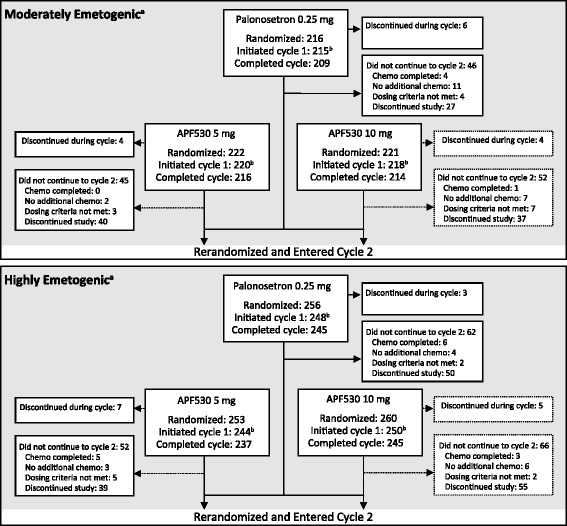
Patient disposition of the overall population in the randomized, double-blind, noninferiority phase III trial (chemotherapy cycle 1). ^a^According to Hesketh criteria [5]. ^b^Safety population. (From Raftopoulos et al. [19], with permission)

The original phase III trial was conducted between 2006 and 2008 at 103 centers in the United States, India, and Poland. In this post hoc subanalysis, we review data from the subpopulation of patients with breast cancer, compared with the overall study population. In the original study, there were 1395 patients in the safety population (patients who received treatment; 653 MEC, 742 HEC), and 1341 patients in the mITT population (patients who were treated and had postbaseline efficacy data) (Fig. 2). Within the subgroup of patients with breast cancer, 629 patients initiated cycle 1 and were included in the safety population (431 MEC, 198 HEC), 608 of whom were included in the mITT population (423 MEC, 185 HEC). Patient demographics and baseline clinical characteristics of the breast cancer mITT population were similar across treatment arms and across MEC and HEC strata (Table 1). The most common chemotherapy regimens were AC-containing regimens in patients treated with MEC (332 of 423, 78 %) and HEC (132 of 185, 71 %) (Table 2). Six patients in the MEC stratum (2 in each treatment group) and 3 patients in the HEC stratum (1 in each treatment group) discontinued treatment after cycle 1. The primary reason for discontinuation was “lost to follow-up” (6 of 9 patients overall).

**Table 1:** Patient demographic and baseline clinical characteristics

	Moderately Emetogenic Chemotherapy			Highly Emetogenic Chemotherapy		
	APF530 250 mg *n* = 149	APF530 500 mg *n* = 140	Palonosetron 0.25 mg *n* = 134	APF530 250 mg *n* = 60	APF530 500 mg *n* = 67	Palonosetron 0.25 mg *n* = 58
Age, mean (SD), y	53.3 (12.05)	54.3 (11.96)	55.0 (11.24)	50.3 (10.83)	49.8 (9.59)	52.6 (12.66)
Female, *n* (%)	149 (100)	137 (97.9)	132 (98.5)	60 (100)	67 (100)	58 (100)
ECOG PS 0–1, *n* (%)	145 (97.3)	140 (100)	131 (97.8)	59 (98.3)	65 (97.0)	56 (96.6)
Race/ethnicity, *n* (%)						
White or Caucasian	80 (53.7)	83 (59.3)	94 (70.1)	17 (28.3)	35 (52.2)	32 (55.2)
Black or African American	11 (7.4)	15 (10.7)	12 (9.0)	8 (13.3)	3 (4.5)	1 (1.7)
Hispanic or Latino	12 (8.1)	9 (6.4)	6 (4.5)	6 (10.0)	6 (9.0)	5 (8.6)
Asian	42 (28.2)	30 (21.4)	22 (16.4)	25 (41.7)	22 (32.8)	20 (34.5)
Other	4 (2.7)	3 (2.1)	0	4 (6.7)	1(1.5)	0
Hesketh class, *n* (%)						
1–2	0	0	1 (0.7)	0	0	0
3	3 (2.0)	6 (4.3)	6 (4.5)	0	0	0
4	145 (97.3)	131 (93.6)	127 (94.8)	2 (3.3)	3 (4.5)	1 (1.7)
5	1 (0.7)	3 (2.1)	0	58 (96.7)	64 (95.5)	57 (98.3)

*ECOG PS* Eastern Cooperative Oncology Group performance status

**Table 2:** Current chemotherapy regimens^a^

	APF530 250 mg	APF530 500 mg	Palonosetron 0.25 mg
MEC regimens, *n* (%)	*n* = 149	*n* = 140	*n* = 134
Docetaxel-trastuzumab	0	1 (0.7)	2 (1.5)
Doxorubicin	1 (0.7)	1 (0.7)	1 (0.7)
Cyclophosphamide-anthracycline	121 (81.2)	108 (77.1)	102 (76.1)
Cyclophosphamide-docetaxel	7 (4.7)	7 (5.0)	13 (9.7)
5-FU-cyclophosphamide-methotrexate	10 (6.7)	11 (7.9)	5 (3.7)
Docetaxel-epirubicin	4 (2.7)	3 (2.1)	3 (2.2)
HEC regimens, *n* (%)	*n* = 60	*n* = 67	*n* = 58
Cyclophosphamide-doxorubicin	2 (3.3)	3 (4.5)	0
5-FU-cyclophosphamide-anthracycline	29 (48.3)	33 (49.3)	33 (56.9)
Cyclophosphamide-docetaxel-doxorubicin	15 (25.0)	10 (14.9)	7 (12.1)
Carboplatin-docetaxel-trastuzumab	5 (8.3)	5 (7.5)	5 (8.6)
Carboplatin-docetaxel	2 (3.3)	5 (7.5)	4 (6.9)
Carboplatin-paclitaxel	0	4 (6.0)	2 (3.4)
Carboplatin-gemcitabine	1 (1.7)	2 (3.0)	1 (1.7)

^a^Received by 3 or more patients

*5-FU* 5-fluorouracil; *HEC* highly emetogenic chemotherapy; *MEC* moderately emetogenic chemotherapy

### Efficacy

**Fig. 3:**
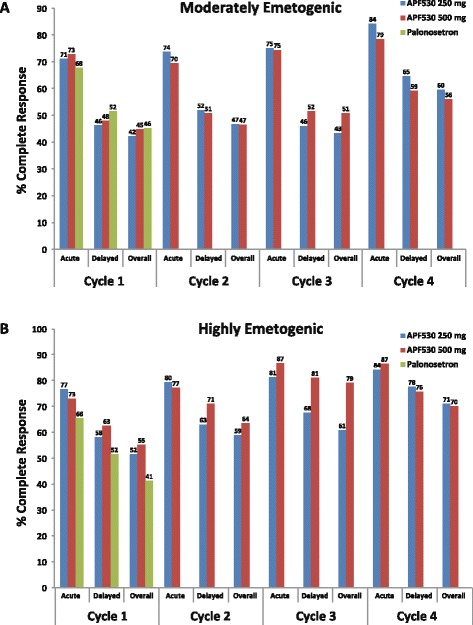
Complete response rates to APF530 250 and 500 mg SC and palonosetron 0.25 mg IV. Graphs show complete response rates in breast cancer patients receiving 4 cycles of (**a**) moderately emetogenic chemotherapy or (**b**) highly emetogenic chemotherapy regimens

**Fig. 4:**
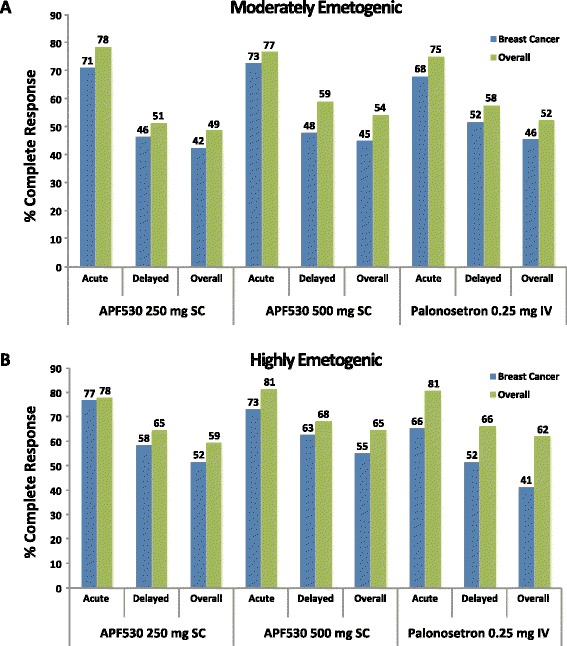
Comparison of complete response rates in cycle 1. Graphs show comparisons between patients with breast cancer and overall study population with (**a**) moderately emetogenic chemotherapy and (**b**) highly emetogenic chemotherapy regimens. *IV* intravenously; *SC* subcutaneously

In the breast cancer subpopulation, CR rates with APF530 250 mg or 500 mg in cycle 1 were not significantly different from CR rates with palonosetron in preventing both acute and delayed emesis with MEC and HEC regimens. There were no significant differences in within-cycle CR rates between APF530 doses during acute and delayed emesis with MEC and HEC regimens in cycle 1 or in subsequent cycles. Complete response rates remained high during the acute phase with both the APF530 250- and 500-mg doses through all 4 cycles (cycle 2, 72 and 78 %; cycle 3, 75 and 84 %; cycle 4, 82 and 85 %, for combined APF530 doses, MEC and HEC respectively), revealing a trend toward higher CR rates in later cycles. High and sustained CR rates were also achieved in cycles 2–4 during the delayed and overall risk periods (Fig. 3). Complete response rates were similar between patients with breast cancer and the overall phase III study population (including breast [45.6 %], lung [18.4 %], and ovarian [10.7 %] cancers) (Fig. 4) [19].

### Safety

The breast cancer safety population (*n* = 629) comprised all patients who were randomized and received study drug. Of these, 75 % experienced an AE, with a similar frequency in each treatment group (Table 3). There were no notable differences in AEs between the breast cancer population and the overall study population. Excluding injection-site reactions, the most common AEs across both the breast cancer and overall populations were fatigue, constipation, and headache. Injection-site reactions occurred across all treatment groups, most commonly during cycle 1, and occurred at a higher rate in the APF530 groups relative to palonosetron. The most frequent injection-site reactions were bruising, erythema, and nodules (Table 3). After completion of cycle 1, there were 3 deaths, 1 in each treatment group, but none was determined to be related to treatment.

**Table 3:** Treatment-emergent adverse events (> 5 %) in any group in cycle 1

Adverse Events^a^	APF530 250 mg SC		APF530 500 mg SC		Palonosetron 0.25 mg IV	
	*n* = 219	*n* = 464	*n* = 211	*n* = 468	*n* = 199	*n* = 463
	Breast Cancer	Overall	Breast Cancer	Overall	Breast Cancer	Overall
At least one adverse event	166 (75.8)	315 (67.9)	161 (76.3)	317 (67.7)	147 (73.9)	313 (67.6)
Preferred term,^b^ *n* (%)						
Asthenia	11 (5.0)	23 (5.0)	10 (4.7)	22 (4.7)	15 (7.5)	30 (6.5)
Constipation	30 (13.7)	62 (13.4)	38 (18.0)	72 (15.4)	25 (12.6)	62 (13.4)
Diarrhea	24 (11.0)	49 (10.6)	25 (11.8)	44 (9.4)	20 (10.1)	39 (8.4)
Fatigue	42 (19.2)	62 (13.4)	37 (17.5)	62 (13.2)	32 (16.1)	52 (11.2)
Headache	24 (11.0)	31 (6.7)	33 (15.6)	47 (10.0)	28 (14.1)	45 (9.7)
Insomnia	12 (5.5)	20 (4.3)	10 (4.7)	25 (5.3)	3 (1.5)	11 (2.4)
Injection-site reactions,^b^ *n* (%)						
Bruising	41 (18.7)	78 (16.8)	54 (25.6)	93 (19.9)	21 (10.6)	41 (8.9)
Erythema	14 (6.4)	33 (7.1)	26 (12.3)	51 (10.9)	9 (4.5)	14 (3.0)
Nodule	12 (5.5)	22 (4.7)	32 (15.2)	50 (10.7)	1 (0.5)	3 (0.6)
Pain	11 (5.0)	16 (3.4)	25 (11.4)	33 (7.1)	3 (1.5)	5 (1.1)

^a^A patient with more than 1 event represented by a given preferred term was counted once within that preferred term

^b^Excludes hematologic adverse events (anemia, leukopenia, neutropenia), abdominal pain, alopecia, and vomiting, which were assumed to be related to chemotherapy

*IV* intravenously, *SC* subcutaneously

After completion of cycle 1, treatment-related AEs occurred in all groups (in 33 % of patients receiving APF530 250 mg; 46 % of patients receiving APF530 500 mg; and 26 % of patients receiving palonosetron). These were generally mild; no patients discontinued because of a treatment-related AE during cycle 1 in the APF530 250-mg and palonosetron groups, but 1 patient discontinued in the APF530 500-mg group because of drug hypersensitivity.

## Discussion

The original analysis of this phase III trial using the entire study population demonstrated noninferiority of APF530 to palonosetron in the control of acute CINV in patients receiving MEC or HEC, and in the prevention of delayed CINV in patients receiving MEC [19]. In this post hoc analysis of the study breast cancer subpopulation, there were no detectable differences between APF530 250 mg and APF530 500 mg SC in within-cycle CR rates in any CINV phase for patients receiving MEC or HEC regimens. Complete response rates for the acute, delayed, and overall CINV periods were sustained across all 4 cycles of chemotherapy with MEC and HEC regimens and at each APF530 dose. Interestingly, CR rates with APF530 tended to increase with subsequent chemotherapy cycles, as was reported for the entire study population [21], although this could be related to the discontinuation of patients whose CINV was not adequately controlled. Comparisons with efficacy data from the entire patient population, which included patients with ovarian, breast, and lung cancers, revealed no notable differences between the breast cancer subset and the overall population in this phase III noninferiority study. Moreover, there were no notable differences in the safety profile of APF530 between the breast cancer population and the overall study population.

The main limitation of this study is that it is a post hoc analysis of a subpopulation from the original study; however, because the phase III trial was one of the largest CINV trials to date, the breast cancer subpopulation was sizeable, at over 600 patients. A possible confounding factor in evaluation of cycle 2–4 results was that some patients had received palonosetron in cycle 1, whereas others had received APF530, prior to re-randomization to APF530 for cycle 2 onwards.

Since the completion of this phase III trial, the criteria used to classify chemotherapy regimens as MEC or HEC have been updated in the ASCO antiemesis guidelines. Importantly, AC-containing regimens, which were considered moderately emetogenic when the original study was conducted, are now classified as highly emetogenic [8], and although a NK-1 receptor antagonist and multi-day corticosteroid in addition to a 5-HT_3_ receptor antagonist are now recommended by the National Comprehensive Cancer Network (NCCN), ASCO, and the Multinational Association of Supportive Care in Cancer (MASCC) for patients receiving HEC, this trial was designed to compare APF530 versus palonosetron as the 5-HT_3_ component. A post hoc subgroup analysis confirmed that reclassification of emetogenicity according to the newer ASCO criteria did not alter initial study conclusions for the overall population [22]. As over 75 % of patients in the breast cancer subpopulation received an AC-containing regimen, this reclassification supports the value of APF530 in providing adequate control of CINV in patients receiving HEC.

Palonosetron is approved for IV administration at a dose of 0.25 mg, the dose used in the current study [23], and has been evaluated in patients with breast cancer. A phase III noninferiority study compared 3 doses of oral palonosetron (0.25, 0.50, and 0.75 mg) with the approved IV dose (0.25 mg) in patients receiving MEC, most of whom had breast cancer. Noninferiority to IV palonosetron was demonstrated by all 3 oral doses in the acute phase and the 0.50-mg oral dose in the overall phase, but none of the oral doses was noninferior in the delayed phase. Addition of dexamethasone generally improved CR rates with the oral doses in the acute and delayed phases. Complete response rates with IV palonosetron were 70, 65, and 59 % during the acute, delayed, and overall phases. These data suggest that the IV formulation of palonosetron is still the preferred option for control of acute and delayed CINV in patients receiving MEC in this population of primarily breast cancer patients [24]. Similar CR rates (65, 68, and 55 %, respectively) were also seen in breast cancer patients receiving MEC in a phase II study of IV palonosetron plus dexamethasone [25]. Given the findings of the original APF530 phase III noninferiority trial and the current breast cancer subanalysis, in which CR rates for APF530 500 mg plus dexamethasone were 73, 48, and 45 % during the acute, delayed, and overall phases following MEC, and 73, 63, and 55 % during the acute, delayed, and overall phases following HEC, APF530 may provide a valuable treatment option for patients with breast cancer, particularly for the prevention of delayed CINV.

## Conclusion

In conclusion, in this population of patients with breast cancer, APF530 and palonosetron provided similar activity in preventing acute and delayed CINV in patients receiving MEC or HEC, and both APF530 doses displayed persistent activity in the prevention of CINV across 4 cycles of chemotherapy. The single subcutaneous injection of APF530 may provide a convenient alternative to palonosetron IV for CINV prevention. Further post hoc analyses may provide additional insights into the efficacy and safety of APF530 SC, including the effects of sex, age, type of chemotherapy regimen, and prior therapies. Future studies may also investigate the potential value of APF530 in other clinical settings where sustained antiemetic activity is desired, such as in patients receiving multiday chemotherapy, in patients receiving radiation therapy, in patients who are unable to tolerate oral antiemetics, in combination with aprepitant and other NK-1 antagonists, and in the postoperative setting.

## Abbreviations

5-HT_3_: 5-hydroxytryptamine type 3
AC: anthracycline (doxorubicin or epirubicin) and cyclophosphamide
AE: adverse event
ASCO: American Society of Clinical Oncology
CC: complete control
CINV: chemotherapy-induced nausea and vomiting
CR: complete response
HEC: highly emetogenic chemotherapy
IV: intravenously
MASCC: Multinational Association of Supportive Care in Cancer
MEC: moderately emetogenic chemotherapy
mITT: modified intent-to-treat
NCCN: National Comprehensive Cancer Network
NK-1: neurokinin 1
QTc: heart-rate corrected
SC: subcutaneously
t_½_: half-life
TEG-POE: tri(ethylene glycol) poly(orthoester)
t_max_: time to maximum plasma concentration
TR: total response

## References

[1] Ballatori E, Roila F (2003). Impact of nausea and vomiting on quality of life in cancer patients during chemotherapy. Health Qual Life Outcomes.

[2] Schnell FM (2003). Chemotherapy-induced nausea and vomiting: the importance of acute antiemetic control. Oncologist.

[3] Roscoe JA, Morrow GR, Aapro MS, Molassiotis A, Olver I (2011). Anticipatory nausea and vomiting. Support Care Cancer.

[4] Craver C, Gayle J, Balu S, Buchner D (2011). Palonosetron versus other 5-HT(3) receptor antagonists for prevention of chemotherapy-induced nausea and vomiting in patients with hematologic malignancies treated with emetogenic chemotherapy in a hospital outpatient setting in the United States. J Med Econ.

[5] Hesketh PJ, Kris MG, Grunberg SM, Beck T, Hainsworth JD, Harker G (1997). Proposal for classifying the acute emetogenicity of cancer chemotherapy. J Clin Oncol.

[6] Hesketh PJ (2008). Chemotherapy-induced nausea and vomiting. N Engl J Med.

[7] Roila F, Herrstedt J, Aapro M, Gralla RJ, Einhorn LH, Ballatori E (2010). Guideline update for MASCC and ESMO in the prevention of chemotherapy- and radiotherapy-induced nausea and vomiting: results of the Perugia consensus conference. Ann Oncol.

[8] Basch E, Prestrud AA, Hesketh PJ, Kris MG, Feyer PC, Somerfield MR (2011). Antiemetics: American Society of Clinical Oncology clinical practice guideline update. J Clin Oncol.

[9] NCCN Clinical Practice Guidelines in Oncology: Antiemesis—v2.2015. http://www.nccn.org/professionals/physician_gls/pdf/antiemesis.pdf. Accessed 19 February 2016.

[10] Chopra I, Kamal KM (2012). A systematic review of quality of life instruments in long-term breast cancer survivors. Health Qual Life Outcomes.

[11] Montazeri A (2008). Health-related quality of life in breast cancer patients: a bibliographic review of the literature from 1974 to 2007. J Exp Clin Cancer Res.

[12] Perry S, Kowalski TL, Chang CH (2007). Quality of life assessment in women with breast cancer: benefits, acceptability and utilization. Health Qual Life Outcomes.

[13] Georgy A, Neceskas J, Goodin S (2007). Antiemetic care for patients with breast cancer: focus on drug interactions and safety concerns. Am J Health Syst Pharm.

[14] NCCN Clinical Practice Guidelines in Oncology: Breast Cancer—v1.2016. http://www.nccn.org/professionals/physician_gls/pdf/breast.pdf. Accessed 19 February 2016.

[15] Chan VT, Yeo W (2011). Antiemetic therapy options for chemotherapy-induced nausea and vomiting in breast cancer patients. Breast Cancer (Dove Med Press).

[16] Kytril (granisetron hydrochloride) injection for intravenous use [prescribing information]. South San Francisco: Genentech, Inc 2011.

[17] Ottoboni T, Gelder MS, O’Boyle E (2014). Biochronomer™ technology and the development of APF530, a sustained release formulation of granisetron. J Exp Pharmacol.

[18] Gabrail N, Yanagihara R, Spaczynski M, Cooper W, O’Boyle E, Smith C (2015). Pharmacokinetics, safety, and efficacy of APF530 (extended-release granisetron) in patients receiving moderately or highly emetogenic chemotherapy: results of two phase 2 trials. Cancer Manag Res.

[19] Raftopoulos H, Cooper W, O’Boyle E, Gabrail N, Boccia R, Gralla RJ (2014). Comparison of an extended-release formulation of granisetron (APF530) versus palonosetron for the prevention of chemotherapy-induced nausea and vomiting associated with moderately or highly emetogenic chemotherapy: results of a prospective, randomized, double-blind, noninferiority phase 3 trial. Support Care Cancer.

[20] Hesketh PJ (1999). Defining the emetogenicity of cancer chemotherapy regimens: relevance to clinical practice. Oncologist.

[21] Boccia R, Cooper W, O’Boyle E (2015). Sustained antiemetic responses with APF530 (sustained-release granisetron) during multiple cycles of emetogenic chemotherapy. J Community Support Oncol.

[22] Raftopoulos H, Boccia R, Cooper W, O’Boyle E, Gralla R. A prospective, randomized, double-blind phase 3 trial of extended-release granisetron (APF530) vs palonosetron (PALO) for preventing chemotherapy-induced nausea and vomiting (CINV) associated with moderately (MEC) or highly (HEC) emetogenic chemotherapy: does a reanalysis using newer ASCO emetogenicity criteria affect study conclusions? J Clin Oncol. 2014;32(suppl):Abstr 9648.

[23] Aloxi (palonosetron HCl) injection for intravenous use [prescribing information]. Woodcliff Lake: Eisai Pharmaceuticals. 2014.

[24] Boccia R, Grunberg S, Franco-Gonzales E, Rubenstein E, Voisin D (2013). Efficacy of oral palonosetron compared to intravenous palonosetron for the prevention of chemotherapy-induced nausea and vomiting associated with moderately emetogenic chemotherapy: a phase 3 trial. Support Care Cancer.

[25] Brugnatelli S, Gattoni E, Grasso D, Rossetti F, Perrone T, Danova M (2011). Single-dose palonosetron and dexamethasone in preventing nausea and vomiting induced by moderately emetogenic chemotherapy in breast and colorectal cancer patients. Tumori.

